# CBCT-based volumetric assessment of the maxillary sinus in temporomandibular disorder: Integration of morphometric analysis and classical machine learning classification

**DOI:** 10.1371/journal.pone.0343691

**Published:** 2026-03-18

**Authors:** Çağatay Bölgen, Sema Polat, Mahmut Tunç, Hazal Duyan Yüksel, Mahmut Öksüzler, Önder Çoban, Önder Yentar, Esin Özşahin, Pınar Göker

**Affiliations:** 1 Medline Adana Hospital, Department of Interventional Radiology, Adana, Turkey; 2 Çukurova University Faculty of Medicine, Department of Anatomy, Adana, Turkey; 3 Başkent University Adana Vocational School of Health Services, Adana, Turkey; 4 Cukurova University, Faculty of Dentistry, Department of Oral Diagnosis and Maxillofacial Radiology, Adana, Turkey; 5 Bozyaka Education and Research Hospital, Department of Radiology, Izmir, Turkey; 6 Atatürk University, Faculty of Engineering, Department of Computer Engineering, Erzurum, Turkey; 7 Department of Computer Science, College of Information Technology, Western Governors University, Salt Lake City, Utah, United States of America; 8 Baskent University, University Faculty of Medicine, Department of Anatomy, Adana, Turkey; 9 Çukurova University Faculty of Medicine, Department of Anatomy, Adana, Turkey; Thamar University, Faculty of Dentistry, YEMEN

## Abstract

**Objectives:**

This study aims to determine the maxillary sinus volume and surface area values and their relationship in individuals with and without temporomandibular disorders (TMDs) using Cone-Beam Computed Tomography and Machine Learning.

**Methods:**

This retrospective study was performed on 127 subjects, 66 in the control group (41 females, 25 males; mean age 28.35 ± 9.9years) and 61 in the TMD group (54 females, 7 males; mean age 35 ± 12.6years) using dento-maxillo-facial CBCT images. Images were acquired as DICOM files and imported into 3D Slicer (version 5.6.2). The volume and surface area of the maxillary sinus were automatically calculated by the 3D Slicer programme. In addition, automatic prediction was performed using classical machine learning techniques on the dataset obtained in the study.

**Results:**

Maxillary sinus volume was 30.85 ± 10.14 cm^3^ in the control group and 26.97 ± 10.33 cm^3^ in the TMD group. Maxillary sinus volume and surface area were significantly smaller in the TMD group compared to controls. No significant differences were observed between age decades in either group. Furthermore, the results obtained in machine learning showed that gender selection generally improved the results, and the most successful classifier was the Logistic Regression algorithm.

**Conclusions:**

This study demonstrates that TMDs were associated with smaller sinus volume. Furthermore, a machine learning-based model can be used to discriminate temporomandibular dysfunction even when the size of the dataset is small.

## Introductıon

The paranasal sinuses, which develop within the bones of the viscerocranium, are located within the skull and the facial bones. The maxillary sinus, the largest of the paranasal sinuses, is located bilaterally within the body of the maxilla bone and has the alveolar bone and the hard palate at its base [1,2]. Begins development at 17th week of the prenatal period and reaching adult size at around 15 years of age, this anatomical structure contributes to vocal resonance, improves the respiratory function of the nose, regulates the humidity and temperature of inhaled air, plays a role in the production of nitric oxide, and thus supports the immune defense of the nasal cavity, reduces the weight of the skull, and protects the orbits and brain. There is a close relationship between the dimensions of the facial skeleton and the volume of the maxillary sinus, and it is thought to play a significant role in determining the shape of the midface and facial contours [3–5]. The maxillary sinus has the capacity to affect the position of the maxilla in relation to the base of the skull, and the anteroposterior direction of development of the maxilla can be affected. Researchers have determined a considerable correlation between mandibular length and maxillary sinus dimensions [6]. Sinus measurements are influenced by many parameters, such as genetics and environment. Furthermore, facial appearance is determined by the size and shape of the maxillary sinus. Proffit and colleagues have demonstrated that adults with long faces have 2–3 times less occlusal force than adults with typical faces. In patients with hyper-divergent and wide gonial angles, a reduced bite force leads to an increase in sinus volume. Some studies have shown that the volume of the maxillary sinus is negatively affected by cleft lip and palate patients, while other studies have shown that it is affected by different breathing types. Studies in the literature have also shown that maxillary sinus measurements are affected by malocclusion [7–10].

Malocclusion, which affects approximately 56% of the world’s population to varying degrees, is defined as any deviation from the normal alignment and relationship of the teeth and jaws. There are three types of malocclusion: Class I involves tooth irregularities with normal molar relationships; Class II exhibits mandibular retrognathism or maxillary prognathism; and Class III is characterized by mandibular prognathism or maxillary retrognathism [11]. Recent studies have revealed a relationship between the temporomandibular joint and malocclusion. It has been suggested that certain types of malocclusion, such as unilateral posterior crossbite, predispose individuals to temporomandibular joint disorder (TMD) symptoms. Temporomandibular disorders (TMDs) are a group of heterogeneous conditions involving the masticatory muscles, temporomandibular joints (TMJ), and associated structures. The most common signs and symptoms are TMJ sounds, pain in the TMJ area and masticatory muscles, and limited or asymmetric mandibular movement [12,13].

The size, growth, and development of the maxillary sinus have already been measured in various studies using dry skulls, panoramic radiography, cone-beam computed tomography (CBCT), multidetector computed tomography (MDCT), and magnetic resonance imaging [14]. In recent years, CBCT has become a popular method for maxillofacial imaging. Compared to CT scans, CBCT scans are quicker and expose patients to less radiation. CBCT scans also provide repeatable and reliable measurements. Moreover, the increasing adoption and clinical value of three-dimensional imaging modalities in cranio-maxillofacial diagnostics have been well established in the literature [15–18].

The current literature indicates that craniofacial morphology may differ in individuals with temporomandibular disorders (TMD). Guercio-Monaco et al. reported that mandibular deviation was associated with disc displacement, with greater deviation toward the more affected side [19]. Xie et al. found that asymmetric anterior disc displacement was linked to increased mandibular asymmetry and reduced condylar height [20]. Bastos et al. and Shi et al. further demonstrated significant relationships between TMD and facial growth patterns or mandibular structure [21,22]. While these findings highlight notable associations between TMD and craniofacial morphology, causal inferences are not justified. These relationships may reflect: (1) craniofacial features predisposing individuals to TMD, (2) TMD-induced functional adaptations leading to secondary morphological changes, or (3) shared developmental factors, such as malocclusion and facial growth patterns, influencing both conditions. This framework provides a biologically plausible explanation for observed variations in maxillary morphology, including sinus volume, without implying direct causation [19–23].

In recent years, there has been a growing interest in the use of artificial intelligence–based approaches within dentistry and maxillofacial imaging. Machine learning (ML), a major subfield of artificial intelligence, is increasingly employed to analyze data derived from three-dimensional imaging modalities such as CBCT. The ability of classical ML models to detect patterns related to morphometric parameters—including sinus volume and surface area—offers a novel perspective for evaluating multifactorial disorders such as TMD. In this context, the present study not only investigates the relationship between maxillary sinus volume and surface area measurements obtained from CBCT and the presence of TMD, but also examines the capability of classical ML algorithms to discriminate TMD using these quantitative metrics [24–26].

Previous CBCT-based studies have demonstrated that maxillary sinus volume correlates with maxillary and craniofacial proportional measurements, highlighting the multifactorial and developmentally shared nature of craniofacial structures [27]. Furthermore Some serious malocclusion characteristics are sometimes associated with the presence of TMDs, while studies have also shown that the maxillary sinus is associated with malocclusion [9,10,12,13]. Within this context, the present study explores whether a similar association may be observed in individuals diagnosed with TMD, within an observational morphometric framework. Accordingly, this study aims to examine maxillary sinus volume in individuals with and without TMDs. Additionally, it was investigated whether ML could be used to evaluate the prediction of TMDs based on sinus volume. To the best of our knowledge, our study is the first in the literature to evaluate the relationship between sinus volume and TMDs. It is believed that this aspect of the study will fill a gap in the literature, provide a new perspective, and contribute to researchers working in this field.

## Methods

### Samples

This study was carried out on 127 subjects, 66 of whom were in the control group (41 females, 25 males; mean age 28.35 ± 9.9 years) and 61 in the TMDs group (54 females, 7 males; mean age 35 ± 12.6 years). Ethics committee approval was obtained from Çukurova University Faculty of Medicine, Non-Interventional Clinical Research Ethics Committee (Decision No: July 03, 2025/153–13), and the necessary institutional permission was also obtained. As the study was retrospective and all CBCT data were fully de-identified before analysis, the requirement for individual patient consent was waived by the committee. The healthy control group was determined by retrospectively searching the archives of patients who underwent CBCT for any reason (implant, impacted tooth, etc.). Also, the archives of patients who underwent CBCT for the TMD group were retrospectively reviewed (patients from 01.01.2020 to 01.04.2025). Data used in this study were accessed for research purposes between 01/05/2025 and 20/05/2025. During the archive review, over 2,000 images were systematically scanned, and after applying strict inclusion and exclusion criteria, only scans of high diagnostic quality and anatomical suitability were retained for analysis. A total of 127 subjects met the eligibility criteria and were included in the study. TMD diagnosis was established according to the Diagnostic Criteria for Temporomandibular Disorders (DC/TMD) by a trained and calibrated examiner. Diagnosis was based on retrospective chart review and clinical examination records. Patients with various TMD subtypes, including disc displacement and dysfunction, were included. CBCT scans were performed within 6 months of the TMD assessment to ensure temporal relevance between imaging and clinical diagnosis [28]. The inclusion criteria for the TMDs group were as follows: a diagnosis of TMD, no history of surgery in the relevant region, no fracture in the relevant region, no systemic disease, no presence of impacted canines or dental implants, patients with a history of sinus lift procedures, and age between 18 and 65 years. Additionally, subjects with conditions known to alter maxillary sinus anatomy—such as acute or chronic sinus disease, previous sinus surgery, recent dental extractions affecting the posterior maxilla, or congenital craniofacial anomalies including cleft palate—were excluded from the study. On the one hand, the inclusion criteria for the control group were as follows: no history of surgery in the relevant region, no fracture in the relevant region, no systemic disease, and being between 18 and 65 years of age.

### Study design

**Fig 1:**
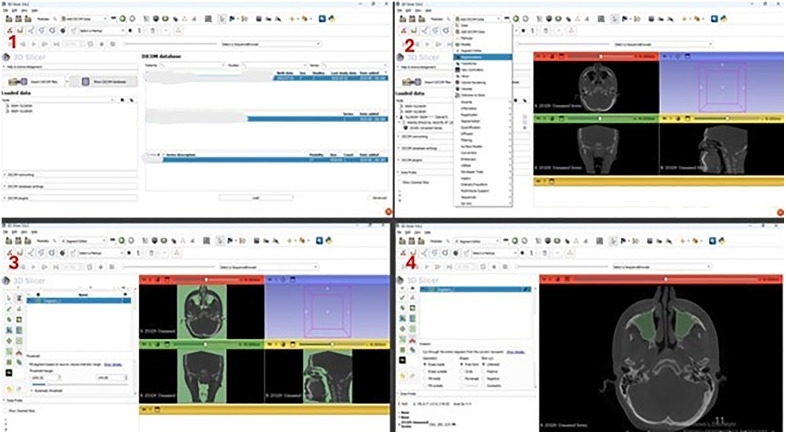
Preparation process for measurement. 1; Uploading the file to the 3D Slicer program. 2; Switching to the segment editor module. 3; Determination of anatomical structure by setting the appropriate threshold. 4; Separation of anatomical structures from other structures by cutting/shaping on the image.

**Fig 2:**
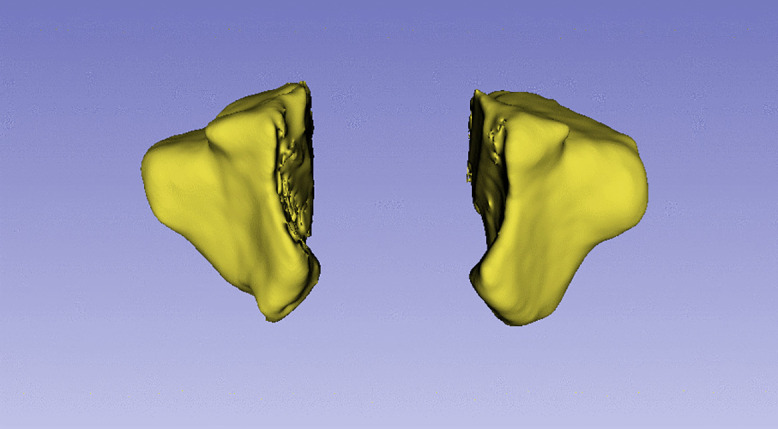
The maxillary sinus was revealed after the necessary procedures that were performed with the 3D slicer program.

This study was conducted with dento-maxillo-facial CBCT images of 127 individuals who were admitted to Çukurova University Faculty of Dentistry for various reasons. All data were fully anonymized before accessing them. Images were taken as DICOM files and imported into 3D Slicer (version 5.6.2). After appropriate threshold adjustment, a 3D model of each image was created, and the maxillary sinus was revealed with appropriate cutting methods. The volume and surface area of the maxillary sinus were automatically calculated by the 3D Slicer programme (see Figs 1 and 2). The decision regarding the inclusion of CBCT images in this study was determined by an experienced dental and maxillofacial radiologist [H.D.Y.] and an anatomist [S.P.]. All measurements were performed by the radiologist [M.Ö.] and anatomist [M.T.], blinded to the group status of each subject to minimize observer bias. In order to minimise intra-observer variability, all measurements were performed randomly by consensus in different sessions at least three weeks apart from the initial assessments. The mean of the two measurements was used for the final value of all measured regions. The intraclass correlation coefficient (ICC) for the two evaluators was 0.805, calculated using a two-way random-effects model assessing absolute agreement for average measures. Threshold values were individually adjusted for each image based on optimal visualization of bone tissue, with minor differences applied between subjects to ensure precise delineation of the maxillary sinus. Segmentation was further refined with manual corrections as needed [29,30]. The volumes and surface areas of the left and right maxillary sinuses were summed to obtain total bilateral values for each subject, which were used in all analyses. In the study, maxillary sinus volume and surface area were evaluated in the control group and the TMDs group. Furthermore, measurements were also evaluated according to age decades in the study.

### Scan parameters

The scan parameters are as follows:

- Planmeca Promax® 3D Mid (Helsinki, Finland)
- exposure parameters
- 90 kV, 10 mA
- scan time: 27 seconds
- utilizing standard resolution mode
- voxel size: 0.4 mm
- field of view (FOV): 20 x 17 cm

All images were evaluated in coronal, axial, and sagittal sections

### Dataset

The dataset contains a total of 127 instances and 4 attributes. The number of target classes is two (1- Control, 2-Patient). While the number of samples in the control (i.e., class 1) group is 66, the number of samples in the patient (i.e., class 2) group is 61. The distribution of 32 male and 95 female individuals between classes in the dataset is as follows (Table 1).

**Table 1:** Distribution of samples (instances) in the dataset in terms of gender and target class.

Class	Gender		Total
	Male	Female	
1	25	41	66
2	7	54	61
Total	32	95	127

These results show that the dataset is sufficiently balanced in terms of the target class, but the distribution between classes is unbalanced in terms of the gender of the individuals.

### Machine learning methods

**Fig 3:**
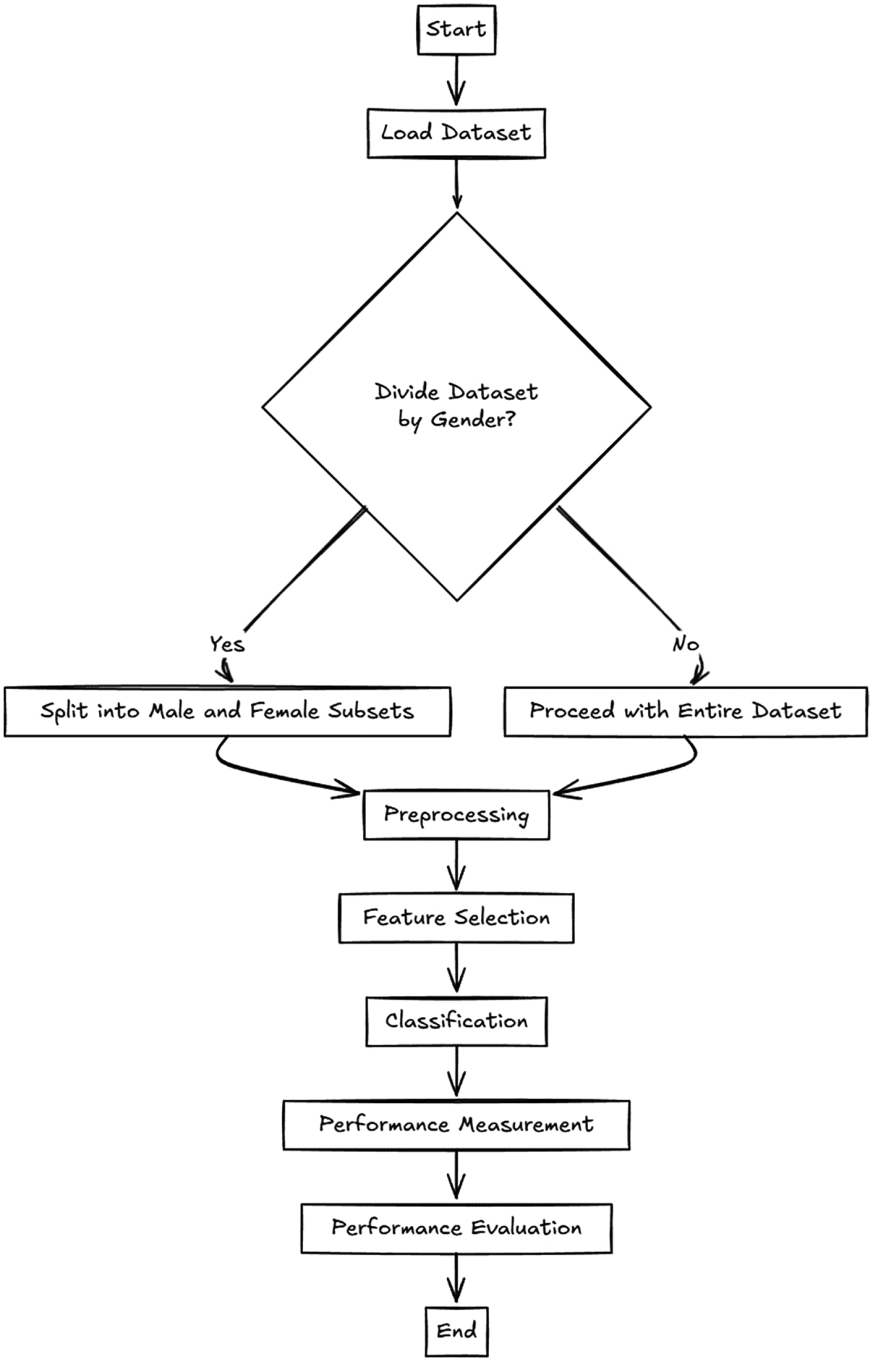
Flowchart of ML pipelines employed in this study.

This study relies on the use of two pipelines depicted in Fig 3. The first pipeline (i.e., baseline) simply follows and employs basic steps on the dataset at hand. The second one, namely a gender-sensitive pipeline, simply divides the data into two disjoint subsets and follows basic steps on these two subsets, including only male and female instances. Note that the basic steps involve preprocessing, optionally feature selection, classification, and finally performance measurement steps that are briefly described under the following headings.

Unlike the baseline pipeline, overall performances are computed by merging the outcomes of learners for male and female instances with their gold (or actual) labels to calculate an overall performance [31,32]. Note that both pipelines use cross-validation to report average performance values across five folds. The pipelines do not involve any aggressive hyperparameter tuning and nested cross-validation process.

### Preprocessing

In this step, imputing and scaling were performed on three attributes. Missing data is imputed by using the interpolate method implemented under the pandas package. Please note that it is selected for imputation since there are only a few missing values in our dataset. On the other hand, it is selected since this method is a straightforward method, assuming a linear relationship between data points. Even though it is often found to be unusual in clinical data, its use in this study does not harm the reliability of our results since there are only a few missing values in the underlying dataset. The scaled attributes, on the other hand, are age, volume, and surface area. Please note that we did not employ any sampling strategy to handle the class imbalance since the skewness between classes is already not too high, as seen in Table 1.

### Classification

In this study, Multi-Layer Perceptron (MLP), Decision Tree (DT), Multinomial Naïve Bayes (MNB), Support Vector Machine (SVM), Random Forest (RF), and Logistic Regression (LR) algorithms [33,34] were used. Please note that all of the classifiers except for the LR and SVM are employed with their default parameter settings, which means that no additional step is applied for hyperparameter tuning. On the other hand, we modified the max_iter and solver parameters of the LR to be 1,000 and ‘liblinear’, respectively. The only modified parameter of the SVM is the kernel, which was selected to be RBF (Radial Basis Function). All other parameters of all classifiers were kept untouched. Please note that SVM and MLP are able to discover non-linear relationships in data, if exists, and therefore are included in the set of classifiers in this study. Another advantage of these methods is that they often work well with continuous feature values.

### Feature selection

In the classification phase, feature/attribute selection was also applied to examine its effect on the results. For this purpose, the wrapper-based SelectFromModel framework implemented in the scikit-learn [35] library was used as an attribute selector. An RF classifier was used as a learner in the feature selector. It should be noted that this selector is intentionally chosen as it can automatically discover the number of attributes to be selected.

### Performance measurement

To measure the performance of the trained models (i.e., classifiers), the metrics Accuracy (Acc), F1-Score (F1-Score), Precision (P), and Recall (R), which are widely used [36,37] in the field of ML, were used. In order to save space and reduce complexity, the mathematical equivalents of the metrics are not given. Stratified cross validation [38] method was used to evaluate and report the performance. Thus, it was ensured that the results were unbiased. Note that the cross-validation is configured to run with five folds. We would like to strongly emphasize that stratification is applied by considering the number of instances across classes [36]. Note that this step is employed to ensure that the proportion of instances in each class is preserved across data splits during cross-validation, so that each training and test set reflects the overall class distribution of the dataset.

We would like to emphasize that all of the ML steps briefly described above were implemented by using the scikit-learn [35] package written in the Python programming language. We feed all of the required parts (packages, classes, learners, etc.) of our implementation with a seed value of 1 to make our results reproducible. Some major packages our implementation relies on, with their versions (provided inside a pair of parentheses following the package name), are as follows: scipy (1.5.0), scikit-learn (1.0.2), pandas (2.0.3), numpy (1.21.6), and matplotlib (3.6.3).

### Statistical analysis

Statistical analyses were performed using SPSS version 22.0 (IBM Corp., Armonk, NY, USA). The distribution of continuous variables was assessed using the Shapiro–Wilk test supplemented by visual inspection of distribution plots. Variables meeting normality assumptions were reported as mean ± standard deviation, whereas non-normally distributed variables were summarized using appropriate descriptive statistics. Between-group comparisons were conducted using the independent samples t-test or the Mann–Whitney U test, depending on distributional characteristics. Categorical variables were analyzed using the chi-square test. Between-group effect sizes were calculated using Cohen’s d for parametric comparisons. Although the proportion of female participants was high in both cohorts, the similarity in sex distribution between the TMD and control groups indicates that sex imbalance is unlikely to introduce systematic bias in the comparison of maxillary sinus measurements, and results were interpreted accordingly. In addition to univariate group comparisons, a multivariable linear regression analysis was performed to assess the independent association between TMD status and maxillary sinus volume. Maxillary sinus volume was entered as the dependent variable, while TMD status (TMD vs. control), age, and sex were included simultaneously as independent variables to adjust for potential confounding effects. Regression coefficients (B), 95% confidence intervals (CI), and corresponding p-values were reported. A significance level of p < 0.05 was accepted for all analyses.

## Results

A total of 127 subjects were included in this study. The TMDs group consisted of 61 subjects, 54 females and 7 males, with a mean age of 35 ± 12.6 years. On the other hand, the control group consisted of 41 female 25 male 66 subjects with a mean age of 28.35 ± 9.9 years. The mean and standard deviations, minimum-maximum values of the measurements from the control group and the TMDs groups are shown in Table 2. According to Table 2, statistically significant differences were found between the groups in terms of maxillary sinus volume and surface area (p = 0.035 and p < 0.001). The average maxillary sinus volume in the TMDs group was 26.97 ± 10.33 cm^3^, while in the control group it was 30.85 ± 10.14 cm^3^. The effect size for the group difference was small-to-moderate (Cohen’s d = 0.38), indicating a modest magnitude of difference between TMD and control participants. Furthermore, the evaluation of the measurements made in the study according to age decades is shown in Table 3. There were no statistically significant differences between age decades in both groups (p > 0.05).

**Table 2:** Comparison of maxillary sinus volume and surface area between TMDs and control groups.

Measurements	Subjects Groups	N	Mean±S.D.	Min	Max	95% Confidence Interval for Mean		P value
						Lower Bound	Upper Bound	
Volume (cm^3^)	Control	66	30.85 ± 10.14	12	76	28.35	33.34	0.035
	TMDs	61	26.97 ± 10.33	11	79	24.33	29.62	
Surface area (cm^2^)	Control	66	83.22 ± 22.02	13	124	77.80	88.63	<0.001
	TMDs	61	64.38 ± 22.09	10	102	58.72	70.04	

**Table 3:** Comparison of maxillary sinus volume and surface area between TMDs and control groups according to age decades.

		Control Group							TMDs Group						
Decade		N	Mean±S.D.	Min	Max	95% Confidence Interval for Mean		P value	N	Mean±S.D.	Min	Max	95% Confidence Interval for Mean		P value
						Lower Bound	Upper Bound						Lower Bound	Upper Bound	
Volume (cm^3^)	Decade 1	12	32.44 ± 9.03	17	48	26.70	38.18	0.843	8	24.87 ± 9.27	14	41	17.11	32.62	0.882
	Decade 2	28	30.53 ± 9.36	12	49	26.91	34.16		16	27.91 ± 8.39	13	43	23.43	32.38	
	Decade 3	15	31.64 ± 14.39	19	76	23.67	39.61		14	25.99 ± 5.43	11	33	22.85	29.13	
	Decade 4	11	28.82 ± 6.63	20	39	24.36	33.28		23	27.65 ± 13.97	11	79	21.61	33.69	
	Total	66	30.85 ± 10.14	12	76	28.35	33.34		61	26.97 ± 10.33	11	79	24.33	29.62	
Surface area (cm^2^)	Decade 1	12	85.70 ± 31.60	13	124	65.61	105.77	0.790	8	55.60 ± 23.02	10	81	36.36	74.85	0.508
	Decade 2	28	83.88 ± 20.24	49	122	76.03	91.73		16	65.60 ± 25.98	10	100	51.75	79.44	
	Decade 3	15	78.23 ± 20.04	32	113	67.13	89.32		14	70.21 ± 10.60	39	80	64.09	76.33	
	Decade 4	11	85.62 ± 18.24	62	111	73.37	97.88		23	63.03 ± 24.14	10	102	52.59	73.47	
	Total	66	83.22 ± 22.02	13	124	77.80	88.637		61	64.38 ± 22.08	10	102	58.72	70.04	

In multivariable linear regression analysis adjusting for age and sex, TMD status was not independently associated with maxillary sinus volume (B = −2.89 cm³, 95% CI: −6.82 to 1.04, p = 0.148). Male sex was independently associated with greater sinus volume (B = 4.87 cm³, 95% CI: 0.44 to 9.30, p = 0.031), whereas age showed no significant association (Table 4).

**Table 4:** Multivariable linear regression adjusting for age and gender.

Predictor	B	95% CI	p-value
TMD status	−2.89	−6.82 to 1.04	0.148
Gender (male)	4.87	0.44 to 9.30	0.031
Age	0.045	−0.11 to 0.20	0.568

### Machine learning results

Methods briefly described in the previous section were employed on the dataset, and results were obtained under different scenarios. The results are given under the following headings, respectively.

### Results without feature selection

In the first stage of the experiments, results were obtained on the scaled dataset without applying attribute selection, and the relevant results are given in the table below (Table 5).

**Table 5:** Results of classifiers – employed on 127 instances without feature selection – with respect to different metrics. The results are obtained with five-fold stratified cross-validation and provided at the 95% confidence level to include lower bound (LB), mean, and upper bound (UB) values.

Classifier	Metric											
	Acc			F1			P			R		
	LB	Mean	UB	LB	Mean	UB	LB	Mean	UB	LB	Mean	UB
MLP	0.483	0.511	0.539	0.330	0.351	0.373	0.249	0.268	0.286	0.483	0.511	0.539
DT	0.440	0.566	0.691	0.434	0.567	0.687	0.441	0.567	0.703	0.440	0.566	0.691
MNB	0.464	0.574	0.684	0.461	0.574	0.680	0.456	0.577	0.706	0.464	0.574	0.684
SVM	0.510	0.582	0.656	0.509	0.582	0.655	0.513	0.582	0.658	0.510	0.582	0.656
RF	0.460	0.598	0.739	0.440	0.597	0.742	0.458	0.597	0.748	0.460	0.598	0.739
LR	0.530	0.637	0.744	0.526	**0.636**	0.737	0.533	0.637	0.764	0.530	0.637	0.744

When the results given in the table above are analysed, it is seen that the highest f1-score of 0.636 is produced by LR. Other classifiers produce lower results, and the MLP classifier shows the lowest performance.

### Results obtained by applying feature selection

From this stage, classifiers were applied together with feature selection. The results obtained are given in the table below (Table 6).

**Table 6:** Results of classifiers – armed with feature selection on 127 instances – with respect to different metrics. The results are obtained with five-fold stratified cross-validation and provided at the 95% confidence level to include lower bound (LB), mean, and upper bound (UB) values.

Classifier	Metric											
	Acc			F1			P			R		
	LB	Mean	UB	LB	Mean	UB	LB	Mean	UB	LB	Mean	UB
MLP	0.463	0.480	0.497	0.293	0.311	0.330	0.214	0.230	0.247	0.463	0.480	0.497
DT	0.456	0.629	0.806	0.449	0.630	0.804	0.467	0.630	0.809	0.456	0.629	0.806
MNB	0.435	0.503	0.572	0.432	0.503	0.566	0.431	0.506	0.585	0.435	0.503	0.572
SVM	0.451	0.582	0.747	0.390	0.582	0.744	0.400	0.582	0.784	0.415	0.582	0.747
RF	0.496	0.614	0.734	0.494	0.614	0.726	0.499	0.614	0.755	0.496	0.614	0.734
LR	0.597	0.669	0.741	0.599	**0.667**	0.734	0.590	0.669	0.760	0.597	0.669	0.741

The results given in the table above show that LR is again the most successful classifier. MLP again lags behind the other classifiers. On the other hand, feature selection improved the performance of LR, RF, and DT classifiers. However, the performance of MLP and MNB classifiers decreased. The performance of the SVM classifier did not change. The highest f1-score obtained was 0.667 with an increase of about 3%. At this stage, it was determined that three of the four attributes (or features) in the current dataset, except gender, were important enough to contribute to the classification process. In other words, it was determined by attribute selection that the gender attribute did not contribute to the classification. The attributes selected in the cross-validation stage are the same in each fold and are three.

### Results obtained with gender-sensitive classification

Finally, gender-sensitive classification was employed on the dataset. Gender-sensitive classification is based on the idea of splitting the very set into two subsets (see Fig 3), males and females, and training and testing two models (which may be the same or different) on these two subsets. It has been observed that this approach improves the results in some cases and can mitigate the performance disparity observed in male and female samples. In this phase of the experiments, the best-case scenario identified in the previous two phases was combined with the gender-sensitive approach. The aim was to save space and reduce complexity.

In other words, at this stage, the gender-sensitive classification approach was applied with attribute selection, and LR was selected as the classifier. The results given in Table 4 show that there is a slightly different improvement in the results (Table 7).

**Table 7:** Results obtained with the gender-sensitive approach (employed on 127 instances and involves the best classifier, LR) across different instance groups (IG). The results are obtained with five-fold stratified cross-validation and provided at the 95% confidence level to include lower bound (LB), mean, and upper bound (UB) values.

IG	Metric											
	Acc			F1			P			R		
	LB	Mean	UB	LB	Mean	UB	LB	Mean	UB	LB	Mean	UB
M	0.613	0.757	0.901	0.537	0.678	0.817	0.476	0.613	0.751	0.613	0.757	0.901
F	0.545	0.643	0.739	0.501	0.613	0.723	0.548	0.673	0.799	0.545	0.643	0.739
O	0.566	0.669	0.774	0.562	**0.668**	0.770	0.573	0.673	0.789	0.566	0.669	0.774

* M and F stand for males and females, respectively. O represents the overall case in which the IG includes both male and female instances.

There is also a difference in performance between males and females. The detection rate is often much higher for male samples with respect to well-known performance metrics. The best attributes selected on male and female samples in each fold during cross-validation are provided in Table 8. Note that each selected feature is provided along with its odds ratio to show the impact of that respective feature on the output.

**Table 8:** Selected features across five folds on male and female instances. Note that the values inside parentheses following the feature name represent odds ratios.

Fold Number	Subset	
	Males	Females
1	Age (1.07), volume (0.99)	Volume (1.00), surface area (0.99)
2	Volume (1.00), surface area (0.99)	Surface area (0.99)
3	Volume (0.99)	Volume (1.00), surface area (0.99)
4	Age (1.02), volume (0.99)	Volume (1.00), surface area (0.99)
5	Volume (0.99)	Volume (1.00), surface area (0.99)

**Fig 4:**
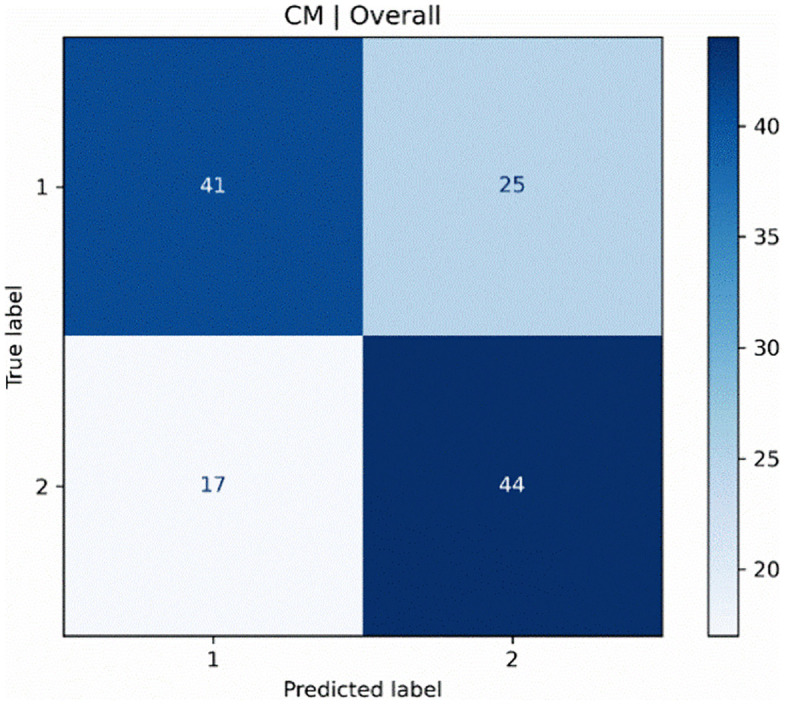
Confusion matrix for the LR classifier used in the gender-sensitive approach. Note that true labels represent the actual labels observed in the data, while predicted labels stand for the predictions of the learner (i.e., LR) at hand. Label values 1 and 2 represent ‘control’ and ‘patient’ classes, respectively.

The attributes given in the table above also reveal a possible reason for the difference. As can be seen from the table, age and volume are generally the discriminative attributes for male samples, while volume and surface area are the discriminative attributes for female samples. Please note that this observation is only valid for our case, and considering our modest results, it is worth noting that practical discrimination of TMD based on sinus morphology alone may not be enough on different but independent datasets. The confusion matrix of the LR classifier was obtained as follows (Fig 4).

**Fig 5:**
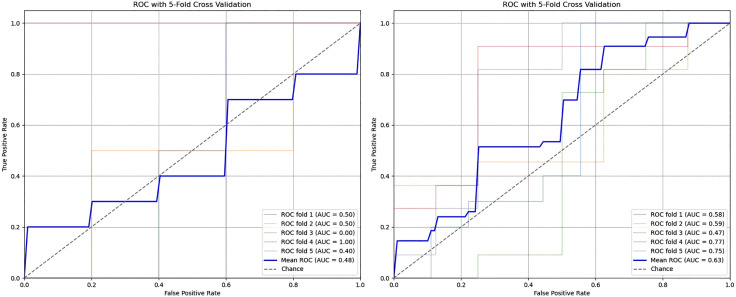
ROC curves across five folds for male and female instances, showing that TPR values are better for females than males.[(for males (left) and females (right)].

As can be seen in Fig 4, 25 examples in class 1 and 17 examples in class 2 were misclassified. The numbers of correctly classified samples for the related classes are located on the diagonal of the matrix and are 41 and 44, respectively. Please note that Fig 4 shows a confusion matrix by merging the predictions and actual labels for male and female subsets. The f1-score of this overall case results in a value of approximately 0.668 (see Table 7). However, this score should be interpreted cautiously since it reflects moderate discrimination at best. Hence, Receiver Operating Characteristic (ROC) curves are also created for both male and female instances using the best pipeline that simulates the gender-sensitive approach armed with feature selection and uses the best classifier, which is LR. The curves for male and female instances are depicted in Fig 5a and Fig 5b, respectively. These curves show that the TPR values are better for female instances, while it is being worse for males. This is an interesting outcome, especially considering male instances on which the pipeline provided an f1-score of 0.667. The reverse is also true for female instances, with an observation that their mean f1-score is lower (0.613) than that of males, but the average TPR value (i.e., 0.63) is higher than the average TPR (i.e., 0.48) value of males. These results indicate that the model is more effective at correctly identifying positive cases (i.e., class 2) among female instances compared to male instances. On the other hand, f1-scores suggest that while the pipeline is better at identifying female instances (higher average TPR), it struggles with precision for female instances compared to males.

### Feature odds ratios

In the previous step, we provided the odds ratios of features for our pipeline that involves both feature selection and the gender-sensitive approach. However, this case does not interpret all available features due to the feature selection. Hence, we also calculated the odds ratios of all existing features in the dataset again with cross-validation. Note that employed the baseline pipeline with the best classifier, LR in this case, and removed both feature selection and gender-sensitive approach to see the odds ratios of all features. We aim to provide a basis for clinical evaluations. The feature odds ratios for the complete set of features are provided in Table 9, which show that age and volume have a stronger effect on the outcome (i.e., control or patient) compared to gender and surface area.

**Table 9:** Odds ratios of the complete set of features. Note that these values are obtained from the baseline pipeline that does not involve feature selection and the gender-sensitive approach.

Fold Number	Feature			
	Age	Gender	Volume	Surface Area
1	0.99681299	1.0629769	1.0000839	0.999445
2	0.99845639	1.06913626	1.0001119	0.99930818
3	0.40137505	1.05448394	1.00010257	0.99942093
4	0.96178644	1.05782933	1.00011215	0.99935254
5	0.99769774	1.05999039	1.00007341	0.99948617

## Dıscussıon

In the present study, the morphological values of the maxillary sinus were measured in individuals with and without TMDs. This allowed for an assessment of whether TMDs affect the volume of the maxillary sinus. Our findings demonstrate that the maxillary sinus volume in individuals with TMDs was 26.97 ± 10.33 cm^3^, while in the control group, this value was recorded as 30.85 ± 10.14 cm^3^. A statistically significant decrease was observed in the TMDs group (p = 0.035). In addition, in the surface measurement, the value was 64.38 ± 22.09 cm² in the TMDs group, while it was 83.22 ± 22.02 cm² in the control group. Again, a statistically significant decrease was observed in the TMDs group (p < 0.001). In this context, although statistically significant differences were observed between groups, these differences correspond to small-to-moderate effect sizes. The functional, diagnostic, or clinical implications of such morphometric differences remain uncertain and should be interpreted within the limitations of observational imaging data. However, regression analysis of these findings suggests that the previously observed group differences reflect not a direct morphological relationship with TMD itself, but rather underlying demographic characteristics, particularly gender distribution. The attenuation of the association after multivariable adjustment does not necessarily negate the presence of a relationship between TMD and maxillary sinus morphology. Rather, it suggests that such a relationship may be context-dependent and influenced by demographic and biological modifiers, particularly sex-related craniofacial differences. Given the heterogeneity of TMD and the complex interplay between craniofacial morphology and functional loading, the absence of an independent association in the adjusted model should be interpreted with caution. Larger, subtype-specific studies incorporating occlusal, cephalometric, and functional variables are required to determine whether specific TMD phenotypes exhibit distinct sinus morphometric patterns. The study also included an evaluation based on age decades. According to this evaluation, no statistically significant differences were observed between decades in either the TMDs group or the control group (p > 0.05). Given the limited sample size within age-decade subgroups, these analyses should be considered exploratory and may be underpowered to detect small effects.

To our knowledge, there are no studies in the literature evaluating the effect of TMDs on the maxillary sinus. In this regard, our study is the first in the literature to investigate how TMDs affect the maxillary sinus. Although there may be many reasons for the lack of studies in this area, in our assessment, the absence of a direct connection between the TMJ and the maxilla has contributed to the lack of studies in this area. However, in our literature review, we observed a connection between the maxillary sinus and TMDs. Studies have demonstrated that malocclusion affects both TMDs and the maxillary sinus. Furthermore, previous studies have shown that TMDs are associated with alterations in craniofacial morphology, particularly affecting mandibular symmetry and condylar structure, which may indirectly influence maxillary morphology [9,10,12,13,19–23].

A comprehensive review of the relevant literature reveals numerous factors that influence maxillary sinus size, including gender, age, tooth loss, malocclusions, different jaw growth patterns, orthodontic treatments, and surgical interventions [1]. Similarly, TMDs have a multifactorial etiology involving various psychological and musculoskeletal factors, and studies on their relationship with other diseases are ongoing [39,40]. One of these is studies that suggest a connection between TMDs and maxillary sinusitis. In a study conducted by Jeon et al. in 2005, it was found that the prevalence of maxillary sinusitis/rhinitis in TMDs patients was found to be 7%. The study revealed that limited mouth opening due to TMDs is a risk factor for both masticatory muscles and sinusitis [41]. In terms of the connection between the TMJ and the maxillary sinus, Dehis and colleagues have found that maxillary sinus volume (MSV) is reduced in Egyptian patients with TMJ ankylosis [42]. In our study, as with other pathologies affecting maxillary sinus volume, a decrease in maxillary sinus volume was observed in TMDs.

The maxillary sinus plays a critical role in the structure and function of the craniofacial complex. Understanding the relationship between maxillary sinus volume and the surrounding craniofacial structures provides meaningful insights in various clinical situations, such as orthodontic treatment planning, dental implant treatment, and treatment of sinus pathologies [2]. In the literature, the point A-Nasion to Nasion-point B plane angle (ANB) and the Sella-Nasion to Nasion-point A plane angle (SNA) are commonly used for cephalometric maxilla-mandible and maxilla-facial analysis. The ANB angle is a measurement that is used to determine the relative positions of the maxilla and mandible in the sagittal plane, while the SNA angle is a measurement taken between the maxilla and the anterior cranial base. The sagittal skeletal groups were classified into three distinct categories according to ANB angles (normal value 2° ± 2°). Class I was designated as 0 < ANB < 4, Class II as ANB > 4, and Class III as ANB < 0. The ANB angle is a crucial element in determining the skeletal pattern/malocclusion [1]. In a study in which Shrestha and colleagues evaluated maxillary sinus volume in different patterns, they showed that the skeletal class II group had significantly larger maxillary sinus volume than the class III group (P < 0.05) and that the high angle group tended to have the largest maxillary sinus volume within the vertical skeletal group [43]. On the contrary, the study conducted by Saccucci et al. found no statistical difference between the classes in terms of maxillary sinus volume. However, they still observed that the maxillary sinus volume of skeletal Class II was significantly larger than that of skeletal Class III [44]. Various implications in dentistry have been found in these results. When a maxillary sinus is large, such as in Class II, High-angle, and males, special care is needed. Special attention is required for orthodontic movement of teeth in the maxillary posterior region. Space closure for missing maxillary posterior teeth through the maxillary sinus is difficult, according to case reports. The use of light forces is recommended for a successful outcome, but this approach lengthens the treatment time. Therefore, awareness of the anatomical relationship between the maxillary sinus and posterior teeth can allow for the prevention of complications in endodontic or oral surgery procedures [45–47].

As indicated by extant literature, an association has been demonstrated between skeletal features and TMJ disc displacement. It has been suggested that skeletal Class II, a retrognathic mandible, and a hyperdivergent growth pattern are generally associated with TMJ disc displacement. In addition, the severity of these skeletal abnormalities seems to be associated with the severity of joint problems [48]. Furthermore, there are studies in the literature that evaluate the relationship between facial morphology and TMD signs and symptoms detected by imaging or clinically in adults. The findings indicate a relationship between lower facial structure (e.g., a hyperdivergent profile tendency) and signs and symptoms of TMDs [23,49,50]. In another study, Flores-Mir et al. evaluated the relationship between craniofacial development and TMJ disc in 79 subjects in Canada in 2006. According to the study findings, TMJ disc anomalies were associated with a decrease in the forward growth of the maxillary and mandibular bodies and a decrease in the downward growth of the mandibular ramus [51]. In a study evaluating maxillary sinus surface area and malocclusions, it was found that in skeletal Class III malocclusions, the height and surface area of the maxillary sinus were significantly larger than in Class I and Class II malocclusions [9]. The literature reports that the morphometric values of the maxillary sinus are affected by malocclusion, and that TMDs are also affected by malocclusion [9,43,44,48]. Malocclusion and craniofacial development have been considered to be a common cluster. In this study, which examined whether the two conditions influence each other, it was found that the maxillary sinus volume and surface area decreased in subjects with TMDs.

Automatic prediction was performed using classical ML techniques on the dataset obtained in the study. The results show that feature selection generally improves the results, and the most successful classifier is LR, whereas the gender-sensitive approach can only improve the results slightly and is not able to mitigate performance disparity between male and female instances. This is due to the fact that the male and female samples do not differ significantly in terms of other attributes. On the other hand, another reason could be that the samples in the dataset may not be labelled correctly. From the viewpoint of learners, the use of a small dataset causes some complex learners, like SVM, to have poor performance. This is because such learners often need a lot of data to learn underlying parameters. Another point to touch on is overfitting in ML side. Fortunately, the pipelines are employed with stratified cross-validation and feature selection that help to obtain unbiased results and mitigate the possible risk of overfitting. Speaking on the predictive power, on the one hand, it is possible to state that classical statistical modelling (i.e., multivariate regression) remains the primary inferential tool, with ML serving a complementary role. Nevertheless, we would like to strongly emphasise that ML models are incorporated into this study primarily with a focus on showing this feasibility for this task, not highlighting their diagnostic power.

The present study has some limitations. Firstly, the study was conducted retrospectively and from a single center which introduces several limitations like a lack of external validation, centre-specific demographic characteristics, limited variation in clinical practice, and so on. A further limitation of the study is the limited generalizability of the findings, as the data were collected from a single center and the participants were from a specific ethnic background and age range. In future studies, morphological changes that occur with the progression of the disease can be recorded after the diagnosis of TMDs. In addition, a lack of malocclusion type control may limit the interpretation of the observed differences. Another limitation is that detailed information about the individuals participating in the study was not included. Another limitation is the lack of detailed occlusal and cephalometric variables, which restricts the ability to account for craniofacial relationships that may influence maxillary sinus morphology. In future studies, respiratory type, detailed information on TMDs, and cephalometric measurements such as SNA and ANB could be included. Moreover, while the CBCT acquisition parameters were appropriate for clinical imaging, the use of a 0.4-mm voxel size may reduce the sensitivity of surface area measurements, particularly when evaluating subtle morphometric variations. ML methods employed in this study are hypothesis-generating and have not undergone external validation, which is crucial to ensure that the underlying models perform well in real-world settings beyond the data they were trained on. As such, this aspect is another limitation of this study, and testing the underlying models on different but independent datasets, using cross-institutional validation, may help to overcome it. Unfortunately, creating and/or accessing real-world data is often very challenging and therefore makes external validation difficult with respect to the aforementioned precautions. One additional limitation is the potential implicit residual confounding related to gender imbalance that found not to help mitigate the performance disparity between male and female instances. This is because gender has a correlation to other features, and therefore, the model at hand may attribute differences to sinus morphology that are actually driven by gender-related anatomy, not TMD status.

In conclusion, to our knowledge, our study is the first to evaluate the effect of TMDs on maxillary sinus volume. We found that TMDs were associated with smaller sinus volume. However, no statistically significant differences were observed between age decades. Our findings indicate that while group-level differences in maxillary sinus volume can be observed between individuals with and without TMD, these differences are not robust to adjustment for basic demographic factors. This underscores the need for carefully controlled, phenotype-specific investigations before sinus morphology can be considered a meaningful correlate of TMD. In addition, the data in this study were analysed using ML approaches. The results indicate that the obtained performance is modest (i.e., f1~≈ 0.66–0.67), and therefore, it is not yet at a level that would support strong conclusions or immediate clinical application. Given the limited size of the dataset, the performance of the ML pipelines should be interpreted with caution. Nevertheless, these pipelines demonstrate the capacity to achieve improved results when trained on larger and more diverse datasets, suggesting potential usefulness for supporting the diagnosis of the target group in future studies. Accordingly, the findings of the present study should not be directly extrapolated to clinical decision-making. Rather than providing evidence for diagnostic or functional relevance, the observed differences should be considered morphometric and hypothesis-generating, highlighting the need for further controlled and phenotype-specific investigations. Taken together, these findings contribute to the existing literature by providing systematically obtained data on maxillary sinus volume in individuals with and without TMD, and by offering a reference framework for future studies investigating craniofacial morphology in more controlled and phenotype-specific contexts.

## Supporting information

S1 File tmj- max sinus spss data. (SAV)
